# Nicotine induced ototoxicity in rat cochlear organotypic cultures

**DOI:** 10.1515/tnsci-2020-0191

**Published:** 2021-10-26

**Authors:** Yi Zhao, Yue Liang, Chunchen Pan, Xiaomin Tang, Yuxuan Sun, Chenyu Xu, Jiaqiang Sun, Jingwu Sun

**Affiliations:** Department of Otorhinolaryngology-Head and Neck Surgery, The First Affiliated Hospital of USTC, Division of Life Sciences and Medicine, University of Science and Technology of China, Hefei, Anhui, 230001, People’s Republic of China

**Keywords:** nicotine, cochlea, hair cells, ototoxicity, organotypic culture

## Abstract

Epidemiological evidence has shown that smoking is associated with an increased risk of hearing loss. However, the underlying mechanisms regarding the impact of nicotine on the cochlea remain unclear. This study aimed to investigate the cytotoxic effects of nicotine on cochlear cells using cultured cochlear basilar membranes. Cochlear basilar membranes were isolated from newborn rats, cultured, and treated with 1–100 ng/mL nicotine for 48 h. Cuticular plates and stereocilia bundle staining were used to evaluate hair cell (HC) loss. Spiral ganglion neuron and acoustic nerve fiber staining were assessed to evaluate cochlear neural injury. Scanning electron microscopy and transmission electron microscopy imaging were employed to examine cochlear ultrastructural changes. Our results showed that compared to spiral ganglia and nerve fibers, HCs are more susceptible to nicotine-induced toxicity. HC loss was more severe in the basal turn than in the middle and apical turns, while nerve fibers and spiral ganglion cells were morphologically maintained. Ultrastructural changes revealed disordered and damaged stereocilia, swelling and decreased mitochondrial density, swelling, and degranulation of the endoplasmic reticulum. Our results suggest that nicotine causes HCs’ degeneration and loss and may have implications for smoking-related hearing loss.

## 1 Introduction

Epidemiological evidence has shown that smoking is associated with an increased risk of hearing loss [1–3]. Smoking has been reported to worsen auditory thresholds at high frequencies, lower response levels in transient evoked otoacoustic emissions, and increase the incidence of tinnitus [1,4,5], suggesting that smoking might be associated with cochlear injuries.

As the primary pharmacological component of tobacco, nicotine acts on various tissues and organs, including the brain and blood vessels [6]. Several studies have been conducted on different dimensions of nicotine-induced ototoxicity, including assessing its effects on auditory function and development [7,8]. A previous *in vivo* study demonstrated that nicotine administration caused damage to outer hair cells (OHCs), disorganization of stereocilia, and expansion of the surrounding supporting cells [9]. However, it has not yet been entirely established that nicotine undermines the cochlear HCs and the relevant afferent neural structure. Therefore, this study aimed to investigate the characteristic injury patterns of cochlear HCs and the relevant afferent neural structure that is affected using nicotine in cochlear organotypic cultures.

## 2 Methods

### 2.1 Animals

Newborn (postnatal 2–3 days, 16 rat pups of both genders with 32 cochleae in total) Sprague Dawley rats were maintained in the animal laboratory of the First Affiliated Hospital of USTC.


**Ethical approval:** All experimental procedures were approved by the ethics committees of University of Science and Technology of China, 2021-N (A)-032 and were performed under the Guide for the Care and Use of Laboratory Animals for Research Purposes.

### 2.2 Cochlear organotypic culture and nicotine treatment

Cochlear basilar membrane culture was performed as previously described [10]. Briefly, anesthetized rat pups were decapitated, the cochlea was separated, and placed in Hank’s balanced salt solution under a dissecting microscope. After exposing the membranous labyrinth, the basilar membrane was quickly dissected and transferred to collagen gel-coated culture dishes with Basal Medium Eagle (BME; Sigma-Aldrich, B1522) containing 10 mg/mL bovine serum albumin, 1% serum-free supplement (Sigma-Aldrich, I1884), 2 mM glutamine (Sigma-Aldrich, G6392), 120 mg/mL glucose, and 100 IU/mL penicillin G. The cultures were maintained under 5% (v/v) CO_2_ at 37°C in a humidified incubator. After overnight incubation, the cultures were treated with different concentrations of nicotine for 48 h. Nicotine solution was prepared from liquid nicotine (N3876; Sigma-Aldrich, St. Louis, MO, USA) and diluted in culture medium to achieve final concentrations. This solution was prepared daily as needed and the original liquid was kept away from air and light at 4°C

### 2.3 Immunofluorescence staining

After treatment, cultured cochlear explants were rinsed and fixed in 4% paraformaldehyde for 15 min. Specimens were subsequently blocked, permeabilized using 5% goat serum, 0.2% Triton X-100, and incubated overnight with anti-Neuronal Class III β-tubulin (1:400, Covance, USA) at 4°C. Next day, the tissues were washed three times and then incubated with Rhodamine conjugated secondary antibody (SA00007-1; Proteintech, China) at a dilution of 1:500 at room temperature for 4 h in darkness. All specimens were labelled with FITC-phalloidin (0.5 g/mL; Sigma-Aldrich), coverslipped, and observed under a fluorescent Leica DMi8 microscope (Leica, Wetzlar, Germany).

### 2.4 Quantification of HCs, spiral ganglion neurons (SGNs), and auditory nerve fibers (ANF)

To assess HC counts, cuticular plates and stereocilia bundles of HCs were stained using FITC-labelled phalloidin (Sigma-Aldrich, P1951, 0.5 g/mL). HC loss was considered to have occurred if either the stereocilia or the cuticular plate could not be identified. OHCs and inner hair cells (IHCs) of each 0.24 mm-long segment were quantified under 400× magnification across the entire length of the basilar membrane, thus from apical, middle, and basal turns [10,11]. SGN density was evaluated as previously described [12]. Three locations in a 141.4 μm × 141.4 μm rectangular area in the middle third of each cochlear culture were quantified. For counting nerve fibers, the average number of nerve fibers per 0.25 mm longitudinal distance in one-third of each cochlear culture was calculated.

### 2.5 Scanning electron microscopy (SEM) and transmission electron microscopy (TEM) observation

Cultured cochlear basilar membranes were prefixed in 2.5% glutaraldehyde overnight and postfixed in 1% osmium solution for 1 h. For SEM observation, specimens were subjected to critical point drying using liquid carbon dioxide and sputter coating using gold-palladium. Images were acquired using a Hitachi S-4800 SEM system (Hitachi, Tokyo, Japan). For TEM observation, specimens were then subjected to dehydration in a graded series of alcohol, embedded with an Eponate 12 kit (Ted Pella, Redding CA, USA), and polymerized at 60 °C for 48 h. Thick sections of 70 nm were cut, mounted on EM grids, stained with 2% aqueous uranyl acetate and lead citrate, followed by washing, drying, and imaging using a JEOL JEM-2100 TEM (JEOL, Tokyo, Japan).

### 2.6 Statistical analysis

All data were statistically analyzed using GraphPad Prism. Data with multiple comparisons were evaluated by one-way ANOVA with *post hoc* comparisons. Differences for single-pair comparisons were analyzed using two-tailed unpaired Student’s *t*-tests. The univariate linear regression model was fitted to explore dose–response relationships relative to different basilar membrane segments, in which the number of HCs was treated as the dependent variable and the log transformed nicotine concentration as the explanatory variable. *P* < 0.05 was considered statistically significant. Data are presented as mean values ± SD or SEM based on the sample size and variability within groups.

## 3 Results

### 3.1 Nicotine induces HC loss in a dose-dependent manner

**Figure 1:**
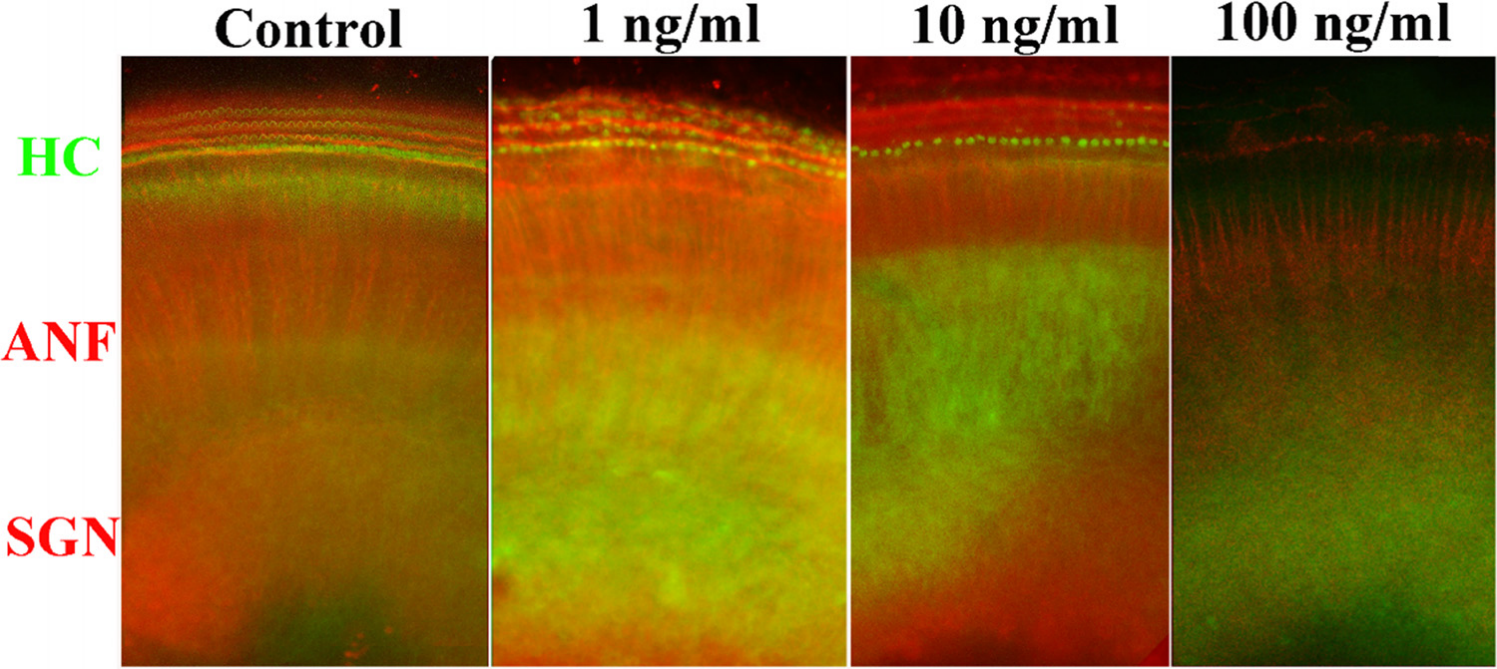
FITC phalloidin staining and rhodamine β-tubulin staining of cochlear cultures treated with different concentrations of nicotine for 48 h. HC stereocilia bundles, SGNs, and ANFs were labelled. HC: hair cell, ANF: auditory nerve fiber, SGN: spiral ganglion neuron, 200× magnifications.

**Figure 2:**
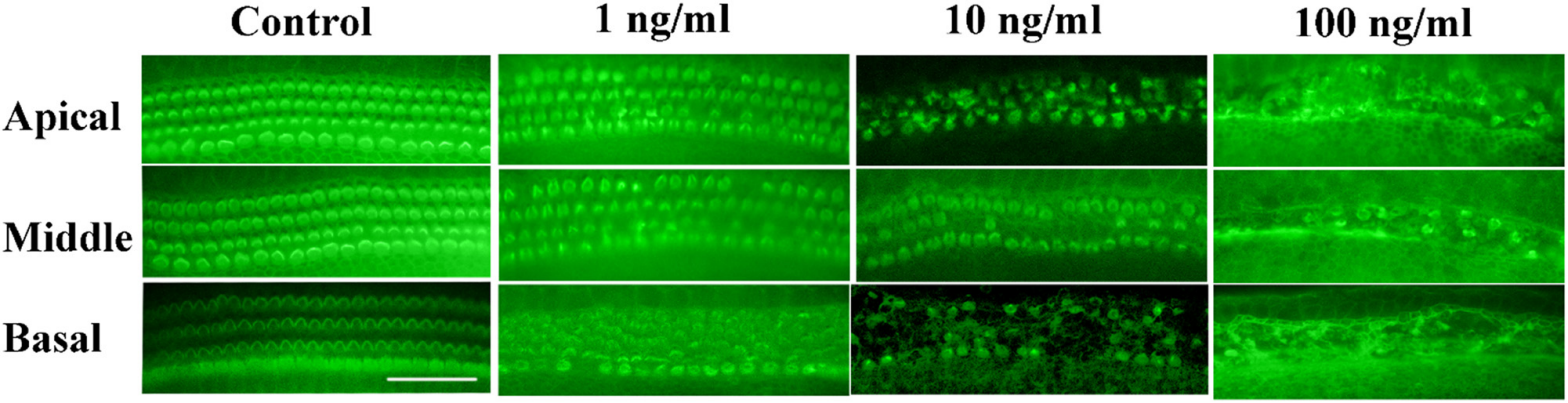
Three segments (apical, middle, and basal turn) of cultured basilar membrane treated with different concentrations of nicotine for 48 h. Scale bar: 50 μm.

The control group exhibited three rows of OHCs and a single row of IHCs arranged in an orderly manner after 48 h of culture, and no HC loss or damage was observed (Figure 1). In contrast, nicotine caused HC loss at a concentration as low as 1 ng/mL. Cochlear explants treated with 1 ng/mL of nicotine exhibited evident damage to stereocilial bundles and mild HC loss, especially at the basal turn (Figures 1 and 2). Higher nicotine concentrations resulted in increased HC damage. When the nicotine concentration increased to 10 ng/mL, the stereocilial bundles of both the IHCs and OHCs collapsed, and loss of HCs was evident at the basal and middle turns (Figure 2). At 100 ng/mL, nicotine severely damaged the cochlear HCs. Few surviving HCs were observed across the whole long basilar membrane (Figures 1 and 2). Stereocilial bundles were either partially or entirely missing, and the cell bodies of HCs were shrunken and disorganized in the surviving HCs. Statistics based on different cochlear segments revealed that HC loss was proportional to the logarithm of nicotine concentration, and the injury started at the base and developed to the apex (Figure 4a and b).

**Figure 3:**
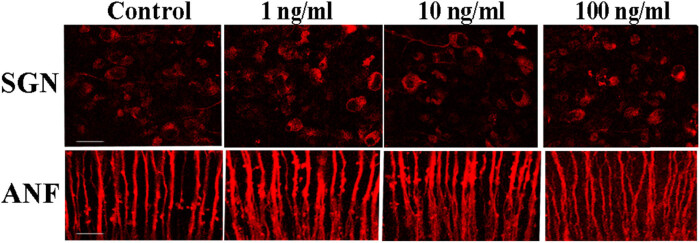
Fluorescence imaging of SGNs and ANFs in the basilar membrane treated with different concentrations of nicotine for 48 h. SGN: spiral ganglion neuron, ANF: auditory nerve fiber, ×400 magnification. Scale bar: 20 μm.

**Figure 4:**
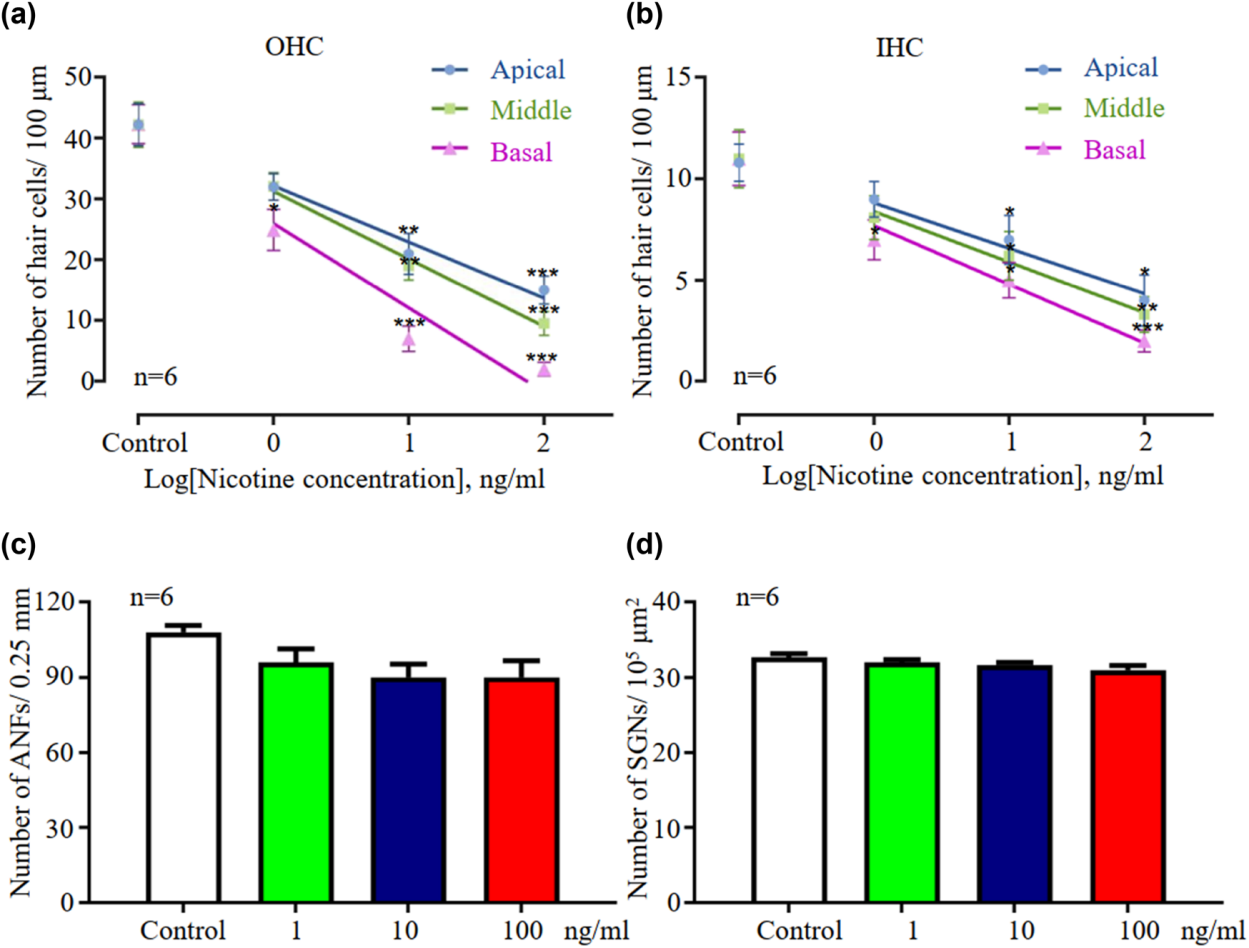
Quantification of nicotine-induced HC loss, changes in SGNs’ and ANFs’ density with different concentrations of nicotine. Number of OHCs (a) and IHCs (b) per 100 μm length of the basilar membrane in three segments (apical, middle, and basal turn). The fitted univariate liner regression model for OHC counts, apical beta coefficients: −9.259, Std. error: 0.556, constant: 32.17, Std. error: 0.681, *P* < 0.001. Middle beta coefficients: −11.120, Std. error: 0.485, constant: 31.25, Std. error: 0.594, *P* < 0.001. Basel beta coefficients: −13.88, Std. error: 0.798, constant: 25.99, Std. error: 0.978, *P* < 0.001. The fitted univariate linear regression model for IHC counts, apical beta coefficients: −2.241, Std. error: 0.197, constant: 8.81, Std. error: 0.242, *P* < 0.001. Middle beta coefficients: −2.500, Std. error: 0.207, constant: 8.394, Std. error: 0.254, *P* < 0.001. Basel beta coefficients: −2.899, Std. error: 0.187, constant: 7.694, Std. error: 0.229, *P* < 0.001. (c) Number of nerve fibers per 0.25 mm longitudinal distance in the one third turn of the basilar membrane. (d) Number of SGN counted in 10^5^ μm^2^ region. *P < 0.05, **P < 0.01, and ***P < 0.01 versus the control group. IHC: inner hair cell; OHC: outer hair cell.

SGNs project the nerve fibers and innervate the HCs of the organ of Corti. Whether cultured under normal conditions or treated with any nicotine concentration for 48 h, both the nerve fibers and SGNs maintained normal morphology (Figures 1 and 3). No pathological changes of any kind were observed. In addition, no significant difference in the quantification of ANFs (one-way ANOVA, *F* = 2.270, *P* = 0.1326) or SGNs (one-way ANOVA, *F* = 2.623, *P* = 0.09) was observed in response to different concentrations of nicotine (Figure 4c and d), suggesting that nerve fibers and SGNs are not significantly damaged by 100 ng/mL nicotine. These results indicated that compared to HCs, spiral ganglia and nerve fibers are less vulnerable to nicotine-induced ototoxicity.

### 3.2 Nicotine induces ultrastructure damages to hair cells

**Figure 5:**
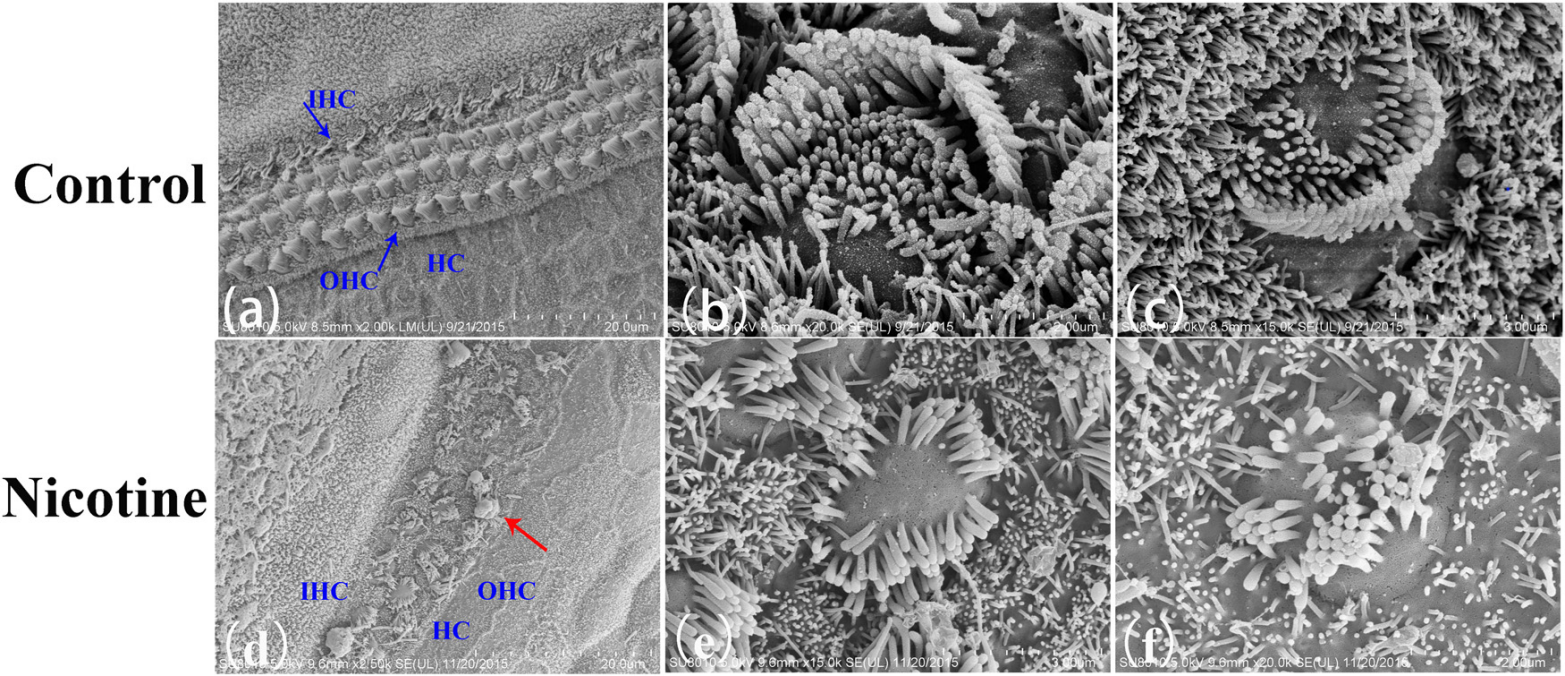
SEM images showing ultrastructural changes in basilar membranes after 1 ng/mL of nicotine treatment for 48 h. (a) Control cultured basilar membrane exhibited orderly arranged OHCs and IHCs. (b) Control OHCs and (c) control IHCs exhibited orderly arranged stereocilia bundles. (d) Cultured basilar membrane treated with nicotine. Injured OHCs detached from the cuticular plate (blue arrow) and vesicular extrusion formed (red arrow). Severely truncated (e) OHC and (f) IHC stereocilia bundles. (OHC: outer hair cell, IHC: inner hair cell, scale bar (a and d): 20 μm; (b, c, e, and f): 2 μm).

SEM revealed that in the control group three rows of OHCs and one row of IHCs were arranged closely and orderly (Figure 5a–c). In contrast, 1 ng/mL nicotine treatment was sufficient to severely damage the basilar membrane, as evidenced by the arrangement of IHCs and OHCs becoming disordered. Renewed supporting cells filled the defect of the HCs, and vesicular extrusion was observed on the surface of the degenerated HCs (Figure 5d). Stereocilia bundles of HCs were severely truncated, and cell bodies were swollen but remained in the cuticular plate [13]. The microvilli of HCs were completely destroyed, while the remaining stereocilia bundles were scattered (Figure 5e and f).

**Figure 6:**
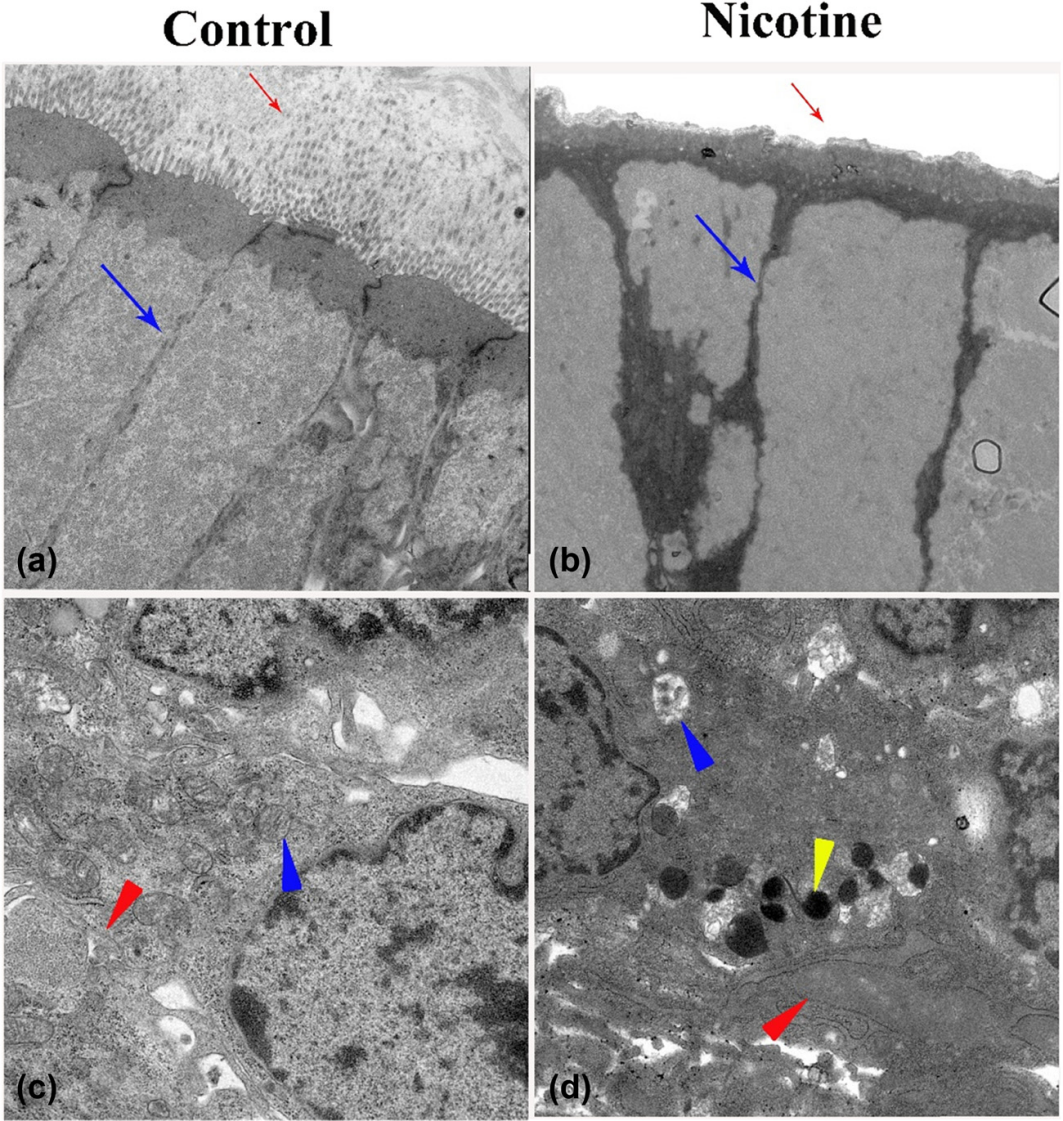
TEM images showing ultrastructural changes in basilar membranes after 1 ng/mL nicotine treatment for 48 h. (a) Stereocilia of OHCs in control cultures are arranged in an orderly manner and filaments are inserted straight. (b) The stereocilial bundles of HCs (red arrow) appear disorderly and collapsed with endocytosis. Tight junction (blue arrow) integrity was disrupted with increased electron density. (c) Mitochondria in control cultures contained multilaminar membrane structures. The endoplasmic reticulum membrane was intact and smooth. (d) The mitochondrial matrices (red triangle) appeared swollen and loose, exhibiting reduced electron density. The endoplasmic reticulum (blue triangle) appeared swollen, and degranulation was observed. Yellow triangle indicates an apoptotic body. (a and b): 1,700× magnification; (c and d): 3,500× magnification.

TEM of the control basilar membranes cultured for 2 days revealed that HCs were arranged tightly, stereocilia were intact, and mitochondria, endoplasmic reticulum, and ribosomal components remained intact (Figure 6a and c). After 48 h of treatment with 1 ng/mL of nicotine, the HCs were arranged loosely, and their tight junctions were disrupted. High electron density was observed deposited at the junction, and the stereocilia were severely damaged (Figure 6b). The nuclear electron density of hair cells decreased, and increased vacuoles were observed in the cytoplasm. The mitochondrial matrices appeared swollen and loose with reduced electron density and broken cristae, indicative of apoptosis. Swollen endoplasmic reticulum and degranulation of rough endoplasmic reticulum suggest the occurrence of an endoplasmic reticulum stress response and multiple apoptotic bodies were observed in the cytoplasm (Figure 6d).

## 4 Discussion

Nicotine is known to act on endothelial, hematological, and epithelial cells and deregulates essential biological processes such as cell proliferation, apoptosis, and angiogenesis [13,14]. Previous *in vivo* studies provided evidence that nicotine administration resulted in HC toxicity, although no measurable hearing impairment was observed [7–9]. However, it remains unclear how nicotine directly influences cochlear HCs and related neural components. The present study demonstrated the deleterious effect of nicotine on the morphology of cochlear organotypic cultures.

We found that nicotine has a larger damaging effect on cochlear HCs than other cell types, and dose-dependent HC loss was observed. With increasing concentrations of nicotine (0–100 ng/mL), the number of hair cells gradually decreased and eventually disappeared. Moreover, nicotine-induced HC loss initiated from the base and developed to the apex, similar to the injury pattern induced by aminoglycoside antibiotics and cisplatin [15,16]. Of note, in the fitted linear regression model, the beta regression coefficients displayed segmental reduction and were more negative from the apical turn to the basal turn, suggesting the increased vulnerability of the basal turn of the cochlea. There are evidence indicating the possible mechanism by which nicotine exerts a pro-apoptotic effect on rat neuronal tissue cultures by binding to nicotinic acetylcholine receptors (nAChRs) at 1, 10, or 100 μM nicotine [17]. Increased intracellular calcium and ROS was detected and believed to lead to neuronal death and apoptosis [14]. The nAChR receptor was also observed in auditory HCs [18]. Therefore, the possibility of receptor-mediated nicotine-induced HC apoptosis deserves further investigation.

Our results suggested that even at high concentrations that destroyed all HCs, nicotine failed to cause visible damage to nerve fibers or SGNs, both of which maintained unchanged morphology and counts. In our study, cochleae from postnatal 2–3 days Sprague Dawley rats were used. Compared to adult animals, auditory nerve structures, e.g., nerve myelin and nerve protrusions, are immature, and surface receptors of auditory neurons are not fully expressed [19,20]. Therefore, these findings cannot fully clarify the responses of SGNs and nerve fibers in adults. Further evidence examining *in vivo* and adult basilar membrane cultures need to be collected.

Evaluation of HC count demonstrated that the surviving proportion of OHCs is less than that of IHCs, indicating that nicotine-induced damage is more severe for OHCs than for IHCs. These findings are consistent with the ototoxic damage caused by other drugs [15]. This differential vulnerability between IHCs and OHCs might be partially attributed to differences in the characteristic transcriptomes of these two cell types [21]. It was found that more robust anti-apoptotic related gene expression was abundant in IHCs, e.g., *BCL* family, while the abundance of mitochondria and increased expression of the pro-apoptotic related receptor, e.g., TNF receptor and nAChR was detected in OHCs, suggesting that these cells are more accessible to apoptosis signals [21]. Therefore, OHCs are more susceptible to damage than IHCs, and changes in the morphology of HCs and stereocilia would directly impair auditory function.

HC stereocilia are composed of embedded bundles of polarized, cross-linked actin filaments arching to the top of the membrane and linked to intracellular filaments. Stereocilia bundles are often lined up in several rows, with V shape formation in IHCs and arc shape formation in OHCs. Staircase-like arranged stereocilia can mechanically activate transduction channels located at the junctions between cells, transforming mechanical energy into electrical signals. Animal experiments have suggested two classical injury patterns of HC stereocilia: stereocilia damage and dislocation of HCs from the cuticular plate [22]. In this study, fluorescent immunostaining revealed that nicotine destroyed HC stereocilia and cuticular plates. SEM also showed that nicotine caused HC detachment and protrusion from the cuticular plates with stereocilia loss and disorder. This pattern of injury was also observed in a previous *in vivo* study with nicotine treatment [9]. Vesicular extrusions in HCs were also observed in our research. Current opinion believes that this type of vesicular extrusion structure is due to the formation of unbalanced endocytosis and efflux processes induced by intracellular sodium overload, indicative of degenerative changes related to apoptosis [23,24]. We speculated that the structures observed in nicotine-treated HCs might suggest HC damage and apoptosis.

TEM revealed that nicotine destroys the normal integral structure of HCs, manifested by disordered morphology of HCs, stereocilia, endoplasmic reticulum, and mitochondria. Furthermore, apoptotic bodies were observed, indicating that nicotine induces HC apoptosis. Stereocilia bundles are susceptible to mechanical and ototoxic drug insults due to their delicate microarchitecture and membrane proteins mediate signal transduction, including ion channels, sensory receptors, and cell adhesion molecules [25,26]. Previous studies have shown that in mammalian cochlear HCs, although tip links at the apex of each stereocilium undergo self-repair after insults, HCs per se fail to regenerate [27,28]. Structural disorders of mitochondria and the endoplasmic reticulum cause abnormal synthesis of actin filaments and degeneration of HC stereocilia. Injured stereocilia impair the transduction of mechanical stimuli into electrical activity, resulting in hearing loss [27].

In summary, we found that *in vitro* culture of the cochlear basilar membrane serves as a good model to investigate nicotine-induced ototoxicity. Compared to spiral ganglia and nerve fibers, HCs are more susceptible to nicotine-induced cochlear damage. HC loss was proportional to the logarithm of nicotine concentration and developed gradually from the base to the apex. Furthermore, ultrastructural observation revealed that nicotine led to disordered and damaged HC stereocilia, mitochondrial swelling and decreased density of mitochondria, endoplasmic reticulum swelling and degranulation, suggesting that nicotine induces HC apoptosis. These findings provide evidence for the mechanisms of nicotine-induced cochlear damage *in vitro*, which may be helpful for the rational design of treatments for nicotine-induced ototoxicity.
